# Distinct NK cell function and gene expression in children with acute lymphoblastic leukemia in remission before and after acute exercise: an exploratory study

**DOI:** 10.3389/fimmu.2025.1625437

**Published:** 2025-08-13

**Authors:** Abel Plaza-Florido, Martin Perlsteyn, Fadia Haddad, Dan M. Cooper, Ronen Bar-Yoseph, Alejandro Lucia, Shlomit Radom-Aizik

**Affiliations:** ^1^ Research Center for Exercise Medicine and Sleep/Pediatric Exercise and Genomics Research Center, Department of Pediatrics, School of Medicine, University of California Irvine, Irvine, CA, United States; ^2^ Institute for Clinical Translational Science (ICTS), University of California Irvine, Irvine, CA, United States; ^3^ Pediatric Pulmonary Institute, Ruth Rappaport Children’s Hospital, Haifa, Israel; ^4^ Department of Sport Sciences, Faculty of Medicine, Health and Sports, Universidad Europea de Madrid, Madrid, Spain; ^5^ Research Institute of the Hospital 12 de Octubre (‘imas12’), Madrid, Spain

**Keywords:** fitness, physical activity, omics, immune function, cancer

## Abstract

**Background:**

Brief bouts of exercise mobilize natural killer (NK) cells and influence their function and gene expression in adults. However, little is known about these effects in children with acute lymphoblastic leukemia (ALL) in remission. This study investigated the effect of acute exercise on NK gene expression and cytotoxic activity (NKCA) in children with ALL in remission.

**Methods:**

Nine B-cell ALL children in remission and 9 age- and sex-matched healthy controls (14.8 ± 1 and 15 ± 1 y/o, respectively; 2 girls per group) performed an acute exercise session consisting of eight 2-min bouts of cycle ergometry at 60% of peak work rate (71 ± 2% of peak oxygen uptake) interspersed with 1-min rest intervals. Circulating NK-cell gene expression profile (RNA-seq) and NKCA (*in vitro* assay) were studied before and after the exercise session.

**Results:**

At baseline, 284 genes were differently expressed in children with ALL compared to controls, and 179 genes were differently altered by acute exercise in the ALL group (p<0.01). At baseline, nine gene pathways related to NK cell function were affected, while following exercise, 28 pathways associated with inflammatory response and cancer were impacted (FDR<0.05). NKCA following IL-2 stimulation was lower both at baseline (p<0.05) and after exercise (p=0.09) in ALL compared to controls. The impaired activity was partially mitigated following exercise but remained lower in ALL compared to controls.

**Conclusions:**

Acute exercise may improve NK cell function in ALL children in remission and has the potential to be used as adjunctive therapy in ALL. The differential gene expression response to exercise suggests that NK cells in ALL may adopt a different molecular strategy to fight infections or tumors.

## 1 Introduction

Acute lymphoblastic leukemia (ALL) is the most common cancer of children and adolescents, with an overall survival rate of 90% in western societies attributed to medical advances (1, 2). Yet, survivors of this malignancy have impaired physical and immune functions (3, 4) and an elevated susceptibility to developing distinct secondary cancers later in life (5), as well as accelerated cellular aging and chronic inflammation (6, 7). Natural killer cells (NK), a type of lymphocyte, play important roles in innate immunity and serve as a first line of defense against pathogens and nascent tumors such as leukemias or lymphomas (8). NK cells show a lower cytotoxic capacity in children with B-cell ALL compared to their healthy peers (9–12). Additionally, the percentage of NK cells in the bone marrow was found to be a prognostic factor in pediatric B- and T-cell ALL, with B-cell ALL (accounting for nearly 90% of cases) (13). While tyrosine kinase inhibitors (TKIs) improve survival in pediatric B-cell ALL (14), they can also modulate NK cell effector function (15, 16). Thus, both the disease *per se* and its treatments can impact NK cell function. Significant progress has been made in treating ALL; nevertheless, many children suffer relapses despite these advances. There is growing data showing that lifestyle factors, specifically levels of physical activity, can have measurable and substantial impacts on how well patients with a variety of cancers respond to treatment (17, 18). Physical activity can exert antitumorigenic effects (17) while also reducing chronic inflammation and improving physical function in survivors of pediatric ALL (19). In adults, NK cells typically show a biphasic response to a single bout of acute exercise (e.g., ≥ 20-60 min of cycling or running at moderate intensities) (17). Thus, an abrupt (i.e., several-fold above baseline) lymphocytosis, including NK cells, occurs during an acute exercise bout and immediately after, with a transient lymphopenia during subsequent recovery wherein previously mobilized cells are redeployed to target tissues (17).

A single bout of acute exercise has also shown to transiently increase the number of circulating NK cells in healthy children (20, 21). Exercise training can increase immune surveillance and might eventually result in higher immune infiltrates into tumors and, potentially, in delayed cancer growth (17). Additionally, there is meta-analytical evidence that a single bout of acute exercise increases natural killer cell cytotoxic activity (NKCA) *in vitro* during 1–2 hours post-exercise in healthy adults (22), but to our knowledge, this remains to be corroborated in the pediatric population. Furthermore, the molecular mechanisms underlying the NK cell responses to exercise need to be explored in depth using state-of-the-art omics technologies (23). In this context, it has been reported that NK cells mobilized into the circulation with a single bout of acute exercise show an anti-tumor transcriptomic profile in healthy adults (24, 25). To the best of our knowledge, the genomic and functional responses of NK-cell to acute exercise in children who are in remission of ALL remain unknown.

This study assessed the effect of an acute exercise session on NK cell genomic response (RNA-seq) and function (NKCA, as assessed *in vitro*) in adolescents with B-cell ALL in remission, compared to age- and sex-matched controls with no history of cancer. Our group’s extensive experience in exercise testing in children and adolescents was essential in ensuring that the exercise input, namely, the relative work intensity and duration, were comparable in both groups. We hypothesized that the NK cells from the participants with B-cell ALL in remission will exhibit a distinct gene expression pattern and functional responses to acute exercise compared to healthy controls. This differential response may reflect an impaired ability to defend against infections and malignancies.

## 2 Methods

### 2.1 Participants and study design

Nine B-cell ALL children in remission stage and nine sex- and age-matched healthy controls with no history of cancer (14.8 ± 1 and 15 ± 1 y/o, respectively; two girls per group) completed two study visits at UC Irvine’s Pediatric Exercise and Genomics Research Center. Exclusion criteria included: i) children with ALL who were unable to exercise or who had accompanying chronic illnesses that would preclude their participation in the study (e.g., cardiovascular diseases) and ii) body mass index (BMI) percentile ≥95th. The Institutional Review Board at the UC Irvine approved the study, and written, informed assent and consent were obtained from all participants and their legal guardians upon enrollment. Participants arrived at the Pediatric Exercise and Genomics Research Center on two separate occasions. The first visit included anthropometric measurements and a standardized aerobic fitness assessment (cardiopulmonary exercise testing – CPET). The second visit included an exercise challenge designed to mimic typical exercise patterns in children. This consisted of eight 2-min bouts of vigorous intensity exercise on a cycle ergometer at a constant work rate (see section 2.3. for details). Blood was drawn before and immediately after the exercise session. Complete blood counts (CBC) and plasma lactate levels were measured. Peripheral blood mononuclear cells (PBMCs) were isolated, and an NKCA assay was performed. NK cells were isolated from PBMCs using a negative magnetic cell separation method and analyzed for gene expression levels using RNA-seq.

### 2.2 Visit 1: anthropometric and cardiopulmonary exercise testing

Standard calibrated scales and stadiometers were used to determine height and body mass. Cardiopulmonary exercise testing (CPET) was done on the first visit using a ramp-type progressive exercise protocol that has been used extensively in children, adolescents, and young adults in our laboratory (24, 26, 27). The participants pedaled on an electronically braked cycle ergometer (VIAsprint^®^ 150p, Ergoline GmbH; Bitz, Germany) until they reached the limit of tolerance indicated either by the participants themselves or by the assessment of the experienced laboratory staff and faculty. Gas exchange was measured breath-by-breath using a Sensor Medics system (Vmax Encore 229; Yorba Linda, CA, USA). Participants were vigorously encouraged to continue pedaling during the high-intensity phases of the test. Peak oxygen uptake ( $\dot{V}$ O_2_peak) was determined as the highest 20-s rolling average in the last minute of CPET.

### 2.3 Visit 2: acute exercise protocol with blood draws

Participants arrived at the laboratory between 7:30–8:30 a.m. after an overnight fast. An indwelling catheter was inserted into the antecubital vein. A baseline sample was taken 30 min after the placement of the catheter and before the onset of exercise. Participants then completed an acute exercise session on a cycle ergometer consisting of eight 2-minute bouts at 60% of the peak work rate achieved in CPET, interspersed with 1-minute rest periods. Gas exchange was measured breath by breath using a metabolic cart. Additional blood samples were obtained immediately after exercise (within one minute). CBCs were obtained by standard methods from the clinical hematology laboratory. Plasma lactate (venous) was measured before and after exercise using an YSI 2300 STAT Plus™ Glucose & Lactate Analyzer (Marshall Scientific; Hampton, NH, USA).

### 2.4 Peripheral blood mononuclear cells isolation and NK cytotoxicity assay

PBMCs were isolated from 20mL of EDTA-treated blood using OptiPrep^®^ Density Gradient Medium (Sigma-Aldrich; St. Louis, MO, USA) and were washed with PBS (calcium- and magnesium-free) and resuspended in 5mL of Complete Medium (RPMI with 1% penicillin-streptomycin and 10% fetal bovine serum). Cell concentration was adjusted to 5 x 10^6^/mL and counted using a Countess II (Invitrogen; Carlsbad, CA, USA). An aliquot of PBMCs containing NK cells was used as effector cells in the NKCA, another aliquot was used to determine the NK cell proportion by flow cytometry, where NK cells were defined as CD3-negative and CD56-positive. The remaining PBMCs were used to isolate NK cells for RNA extraction.

Seven children with ALL in remission and seven sex and age-matched controls with sufficient PBMC yield were included in the NKCA analysis. The NKTEST^®^ kit from Glycotope Biotechnology (Allele Biotechnology, San Diego, CA) was used to evaluate NKCA following the kit instructions. This kit quantifies NKCA using 10,000 green fluorescent K562 target cells incubated with PBMCs as effector cells at ratios of 0:1 and 25:1 for four hours at 37°C. In these NKTEST assays, effector cells were the PBMCs, peripheral blood mononucleated cells containing NK cells, monocytes, and T-lymphocytes. The 0:1 ratio (target cells only) served as a negative control to account for spontaneous cell death during the 4 hours incubation at 37°C. Specific NKCA was calculated by subtracting the control activity from the test sample activity at 25:1. To assess the effect of IL-2 stimulation, effector cells were incubated with interleukin (IL)-2 at 60 units/mL, a concentration known to increase NK cell cytotoxic activity (28). The goal was to compare IL-2–induced cytotoxic activity between children with ALL in remission and healthy controls. Post-incubation, killed target cells were identified by red DNA staining and analyzed via flow cytometry (BD Accuri C6 flow cytometer). NKCA was reported as the percentage of red-stained cells relative to the total green-stained cells. The NKCA assays were performed in duplicates.

### 2.5 NK cell isolation and RNA sequencing

NK cells were isolated from PBMCs using the Miltenyi Biotec NK Cell Isolation Kit (Cat# 130-092-657; Miltenyi Biotech, Bergisch Gladbach, Germany) and the AutoMACS Pro Separator. Non-NK cells were magnetically labeled and depleted (negative selection) to achieve high-purity NK cell preps. NK cell purity was determined by flow cytometry, averaging 88% at baseline (range 80-93%) and 92% (range 88-95%) at peak exercise. The isolated NK cells were washed with PBS and resuspended in Trizol, and RNA was extracted using the Trizol method. The RNA was then treated with DNase and purified using Zymo RNA cleanup columns. RNA concentration was measured with a Nanovue spectrophotometer, and RNA integrity was assessed using an Agilent Bioanalyzer, with RIN scores averaging above 8.0.

Library preparations, RNA sequencing, and data processing were performed at the UC Irvine’s High Throughput Facility, with whole transcriptome sequencing conducted on an Illumina HiSeq platform (~200M X2 paired-end 100 bp reads). The reads for whole transcriptome sequencing were downloaded and analyzed using FASTQC (v.0.11.2), then trimmed using Trimmomatic (v.0.32) with Illumina TruSeq adapter sequences, PHRED quality score 15 and minimum length 20 bases. The trimmed reads were aligned to the Human hg19 reference genome with transcriptome annotation and post-processed using Tophat2 (v.2.0.12), Bowtie2 (v.2.2.3), and Samtools (v.0.1.19). Expression levels were quantified both with fragment per kilobase per million mapped reads using Cufflinks (v.2.1.1) and with raw counts using HTSeq (v.0.6.1p1.) (GEO accession number GSE272928).

### 2.6 Data analyses

An unpaired Student’s *t-*test for between-group comparisons at baseline of anthropometric variables, cardiorespiratory fitness, and exercise intensity was performed. A repeated-measures two-way (group, time) analysis of variance (ANOVA) was used to examine differences for those variables (i.e., lactate, NK cell proportions) pre-post exercise. Non-parametric Wilcoxon and Mann-Whitney U tests were performed to explore NKCA with and without IL-2 stimulation differences in response to exercise. Results were considered statistically significant at p<0.05.

Differential gene expression analysis was performed using DESeq2 (v.1.38.3). Paired design was used to account for samples from the same patient. Multifactor design with interaction terms was applied for group differences, and a p-value cut-off of 0.01 was considered statistically significant. Canonical pathways and interactive network analyses were performed using QIAGEN’s Ingenuity^®^ Pathway Analysis (IPA^®^) (QIAGEN, Redwood City, CA, USA; www.qiagen.com/ingenuity) (29). Canonical pathways with a false discovery rate (FDR) <0.05 were considered statistically significant. The calculation of activation Z-scores in IPA is described elsewhere (29). In addition, interactive gene network analysis was performed based on the connectivity among genes altered by exercise and the top-IPA network with the cut-point of 35 molecules (default settings) and the highest IPA score was presented (30).

## 3 Results

**Figure 1:**
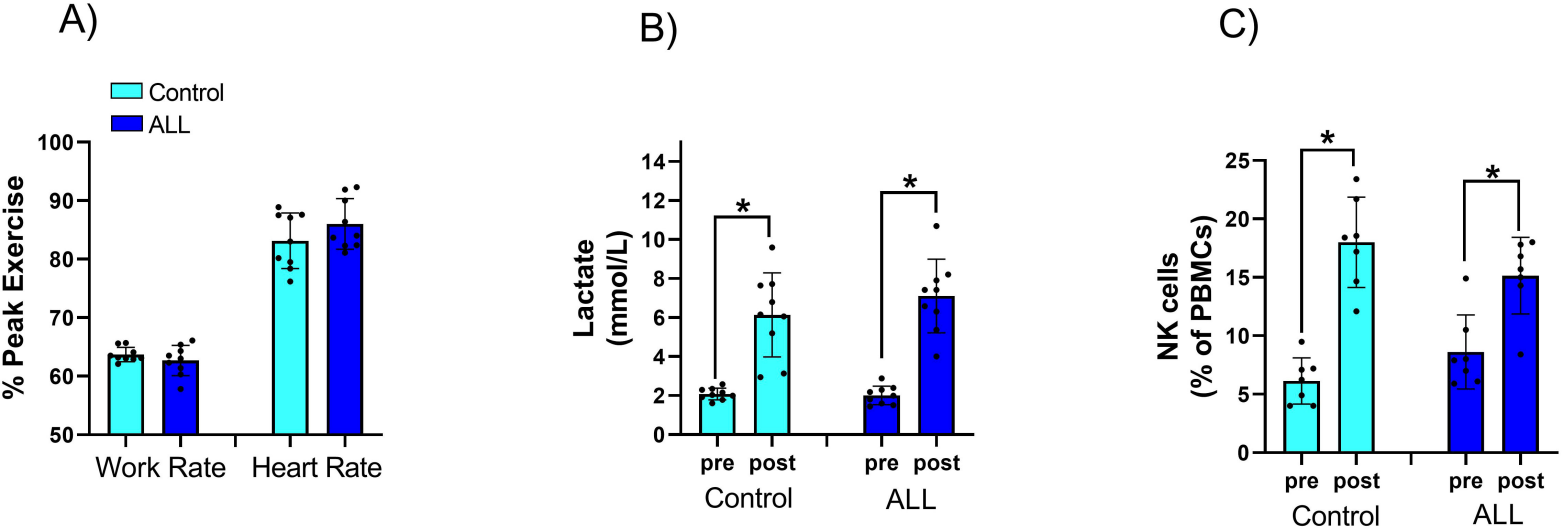
Relative exercise intensity of the exercise session was similar in ALL children in remission and controls **(A)** Blood lactate **(B)** concentration and NK cell proportions **(C)** increased significantly following the acute exercise session in both groups, with no significant group by time interaction effect. Data are mean ± SD and individual data values. *p < 0.05.

**Figure 2:**
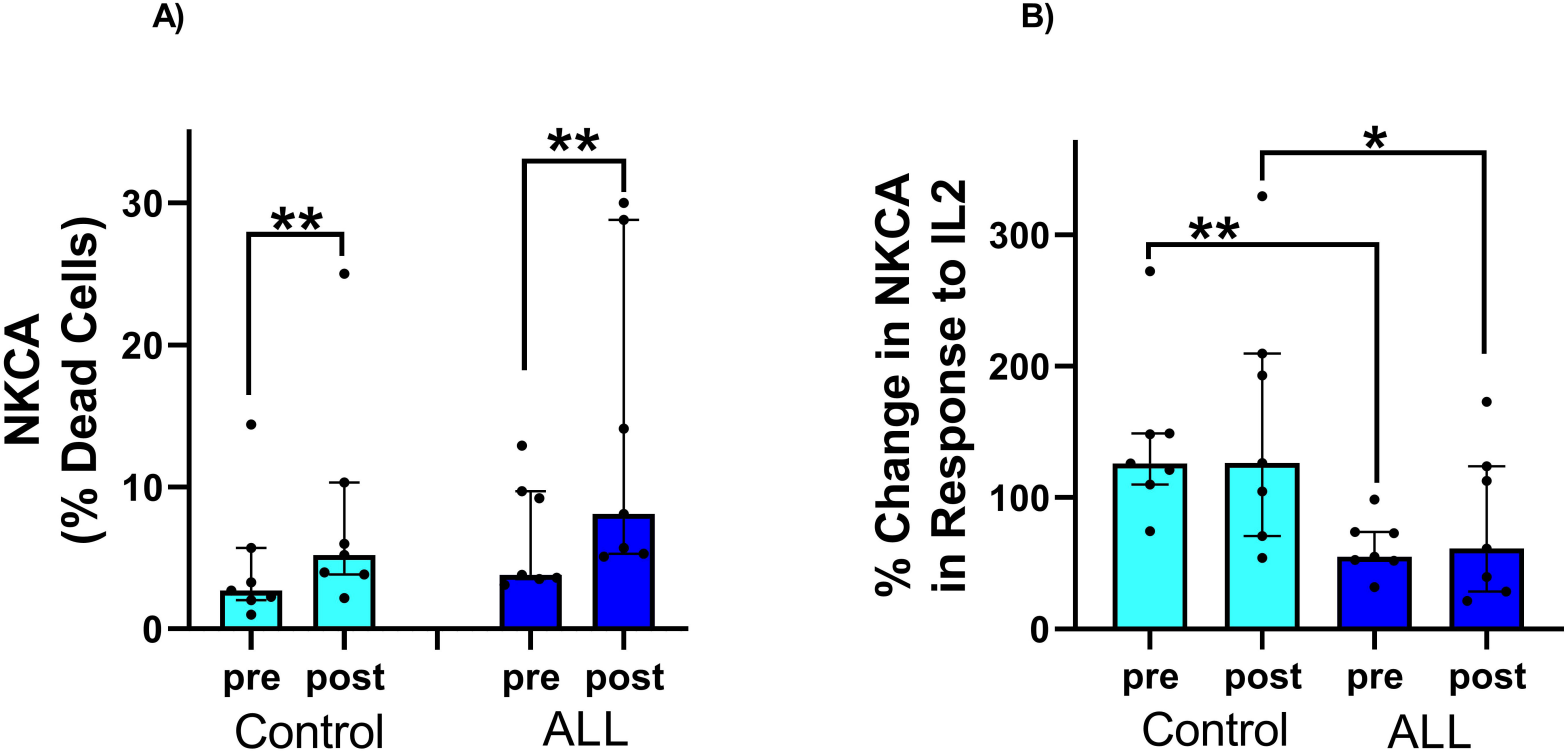
**(A)** Significant increase in NK cells killing activity in response to exercise in both control and children with ALL in remission. The % increase in NKCA in response to exercise was similar in both groups. **(B)** There was a significant increase in NK cell killing activity in response to IL-2 stimulation in both the control group and children with ALL in remission. However, the percentage increase in NKCA in response to IL-2 stimulation was significantly lower in children with ALL in remission compared to the control group, both at baseline and after exercise. Data are median, interquartile range, and individual values. **p < 0.05; *p < 0.1. Abbreviations: ALL, acute lymphoblastic leukemia; CON, control; NK, natural killer; NKCA, NK-cell cytotoxic activity (as assessed *in vitro*).

**Table 1** shows anthropometric characteristics and VO_2_peak in children with ALL in remission and controls, with no statistically significant difference between the two groups (P>0.05). Relative exercise intensity of the exercise session (percentage of peak work rate and peak heart rate) was similar in ALL children in remission and controls (**Figure 1A**). Blood lactate concentration (a surrogate for intense effort) and NK cell proportions increased significantly following the acute exercise session in both groups (P=0.001 for time effect in ALL and controls), with no significant group by time interaction effect (P=0.296 and 0.263 respectively) (**Figures 1B, C**). Both ALL and control groups increased NKCA following exercise (P=0.02 in both groups) with no significant differences between groups (P=0.33) (**Figure 2A**). At baseline, NK cells from ALL children in remission exhibited lower NKCA following IL-2 stimulation compared to controls (average 62% *vs*. 143%, respectively; P=0.001), and after exercise, NKCA increased in both groups but remained lower in ALL compared to controls, (average 80% *vs*. 155%, P=0.09, [**Figure 2B**]).

**Table 1:** Anthropometric variables and fitness.

Variable	ALL (N=9; 2 female)	Controls (N=9; 2 female)	P-value
Age (years)	14.8 ± 0.8	15.0 ± 0.9	0.51
Height (cm)	160.7 ± 4.0	165.9 ± 4.8	0.30
Body mass (kg)	56.8 ± 3.7	60.8 ± 6.2	0.53
BMI (percentile)	63.6 ± 9.9	54.4 ± 11.6	0.88
VO_2_peak (mL/kg/min)	41.0 ± 3.3	47.1 ± 3.2	0.20
Work rate peak (Watts)	150.0 ± 12.8	192.8 ± 2.8	0.13
Heart rate peak (bpm)	196.1 ± 4.7	197.3 ± 2.8	0.84
Peak respiratory Exchange Ratio (VCO_2_/VO_2_)	1.26 ± 0.04	1.25 ± 0.03	0.85

Values are means ± SE. ALL, acute lymphoblastic leukemia; BMI, body mass index; bpm, beats per minute; VCO_2_/VO_2,_ ratio between the volume of carbon dioxide and oxygen consumed; VO2peak, peak oxygen consumption. No significant between-group differences were found.

**Figure 3:**
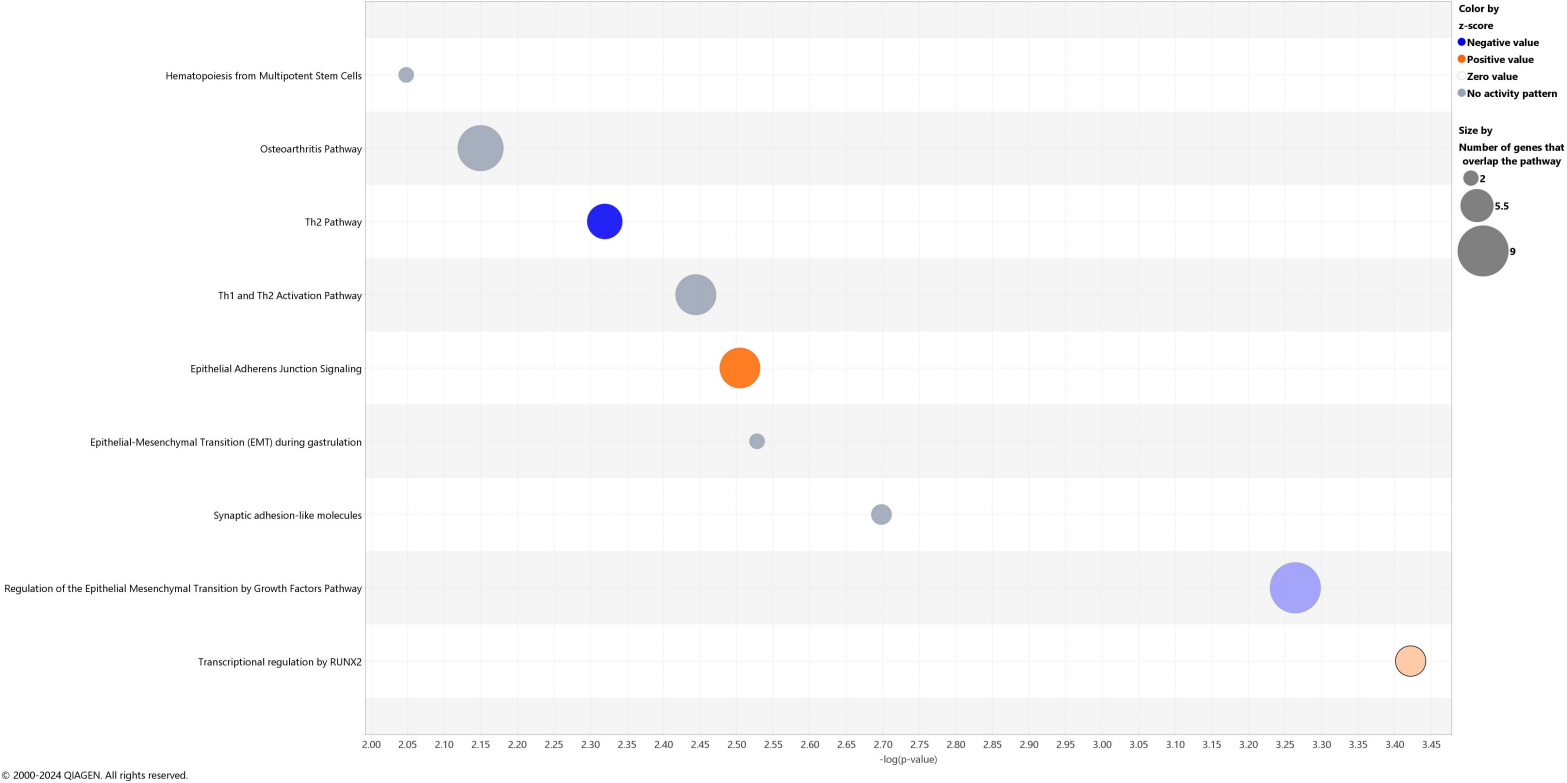
Canonical pathways showing different gene expression patterns at baseline in children with acute lymphoblastic leukemia in remission compared to controls. The blue color represents inhibition and orange activation, and the gray effect is not predicted. The x-axis reflects the p-value, while the y-axis corresponds to the names of the pathways.

At baseline, 284 genes were differentially expressed in NK cells of children with ALL in remission compared to controls (160 genes down-regulated and 124 up-regulated; log_2_FC ranged from –5.56 to 6.19, p < 0.01 [**Supplementary Table S1**). These genes were enriched in 9 gene pathways related to immune function (e.g., hematopoiesis from multipotent stem cells, Th1 and Th2 Activation Pathway, transcriptional regulation by RUNX2; p < 0.01, **Figure 3**). Two pathways (“Th2 Pathway” and “Regulation of the Epithelial Mesenchymal Transition by Growth Factors Pathway”) were inhibited in NK cells from children with ALL in remission compared to controls (Z-score values of activation -0.82 and -0.33 respectively), while two pathways (“Epithelial Adherens Junction Signaling” and “Transcriptional regulation by RUNX2”) were activated in the former (Z-score values of activation 1.13 and 0.45 respectively) (**Figure 3**). Several genes involved in IL-2 family signaling (*HAVCR2*, *IL3RA*, *INPP5J*), Glucocorticoid Receptor Signaling (*ANXA1*, *AR*, *HLA-DQA1*, *IFNLR1*, *IL3RA*, *IL4*, *KRT1*, *MMP1*, *MT-ND3*, *MT-ND6*, *RARB*, *SDHA*), PI3K/AKT Signaling (*IFNLR1*, *IL3RA*, *INPP5J*, *ITGAD*, *YWHAQ*), and molecular mechanisms of cancer (*ADGRA3*, *C5AR2*, *CDKN2C*, *CTNNA1*, *GPR173*, *GPR183*, *IFNLR1*, *IL3RA*, *ITGAD*, *MMP1*, *PTGDR2*, *PTGER3*, *RBL1*) were differently expressed in NK cells from children with ALL in remission compared to controls (**Table 2**, **Supplementary Tables S1**, **S2**).

**Table 2:** Selected genes showing differential baseline expression in NK cells from B-cell acute lymphoblastic leukemia (ALL) patients compared to controls.

Gene pathways	Genes identified in the pathway (log_2_FC ALL *vs* Control)
IL-2 family signaling	*HAVCR2* (0.48), *IL3RA* (-1.15), *INPP5J* (-2.62)
Glucocorticoid Receptor Signaling	*ANXA1* (0.44), *AR* (-1.56), *HLA-DQA1* (-1.24), *IFNLR1* (1.69), *IL3RA* (-1.15), *IL4* (-3.83), *KRT1* (0-1.53), *MMP1* (3.45), *MT-ND3* (-0.54), *MT-ND6* (-1.06), *RARB* (2.34), *SDHA* (0.20)
PI3K/AKT Signaling	*IFNLR1* (1.69), *IL3RA* (-1.15), *INPP5J* (-2.62), *ITGAD* (1.45), *YWHAQ* (0.25)
Molecular mechanisms of cancer	*ADGRA3* (-0.84), *C5AR2* (-1.06), *CDKN2C* (0.49), *CTNNA1* (0.62), *GPR173* (-1.00), *GPR183* (-0.75), *IFNLR1* (1.69), *IL3RA* (-1.15), *ITGAD* (1.45), *MMP1* (3.45), *PTGDR2* (-1.12), *PTGER3* (-1.79), *RBL1* (0.35)

**Figure 4:**
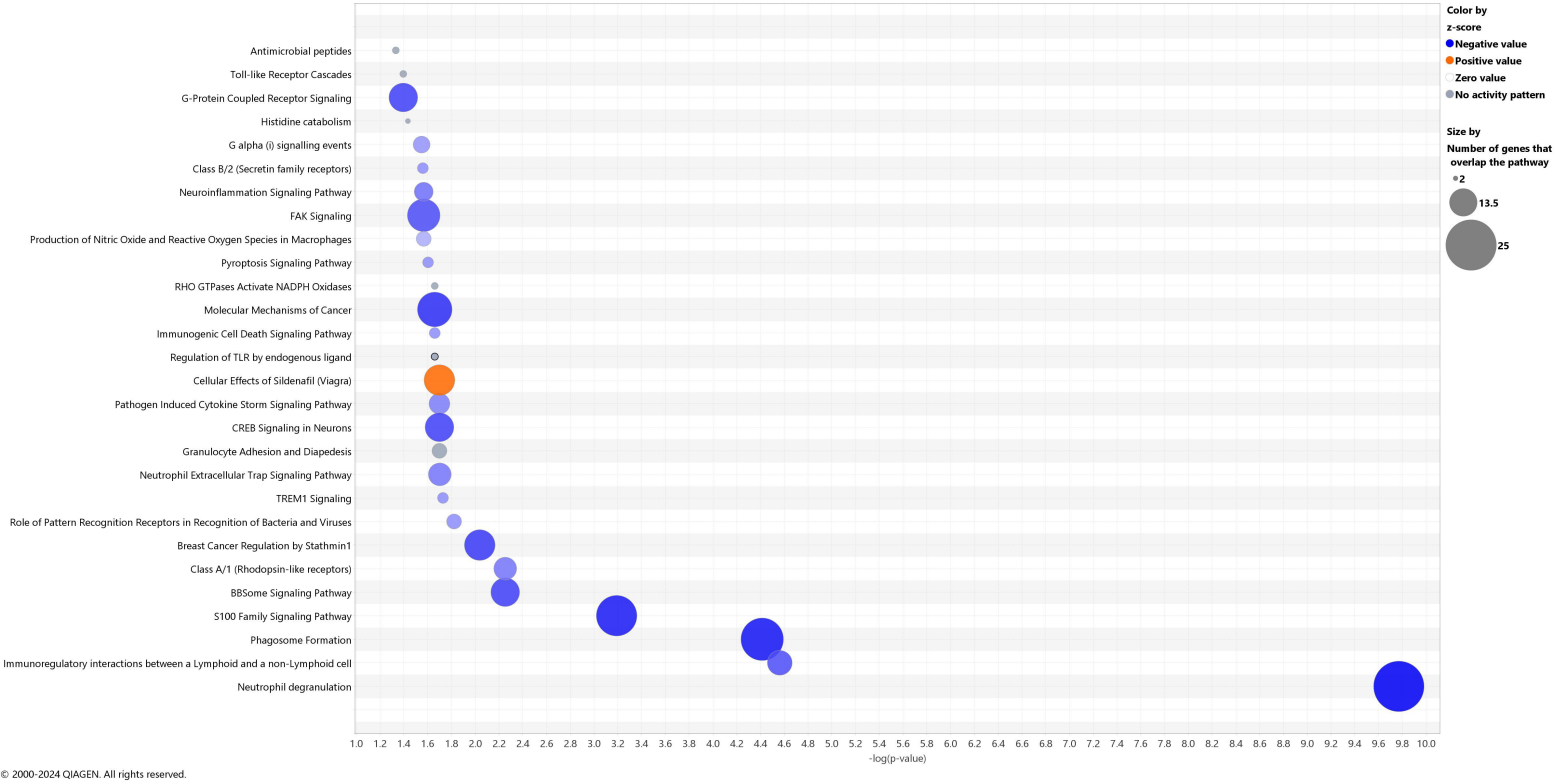
Canonical pathways differently altered by acute exercise in children with acute lymphoblastic leukemia in remission compared to controls. The blue color represents inhibition and orange activation, and the gray effect is not predicted. The x-axis reflects the p-value, while the y-axis corresponds to the names of the pathways.

**Figure 5:**
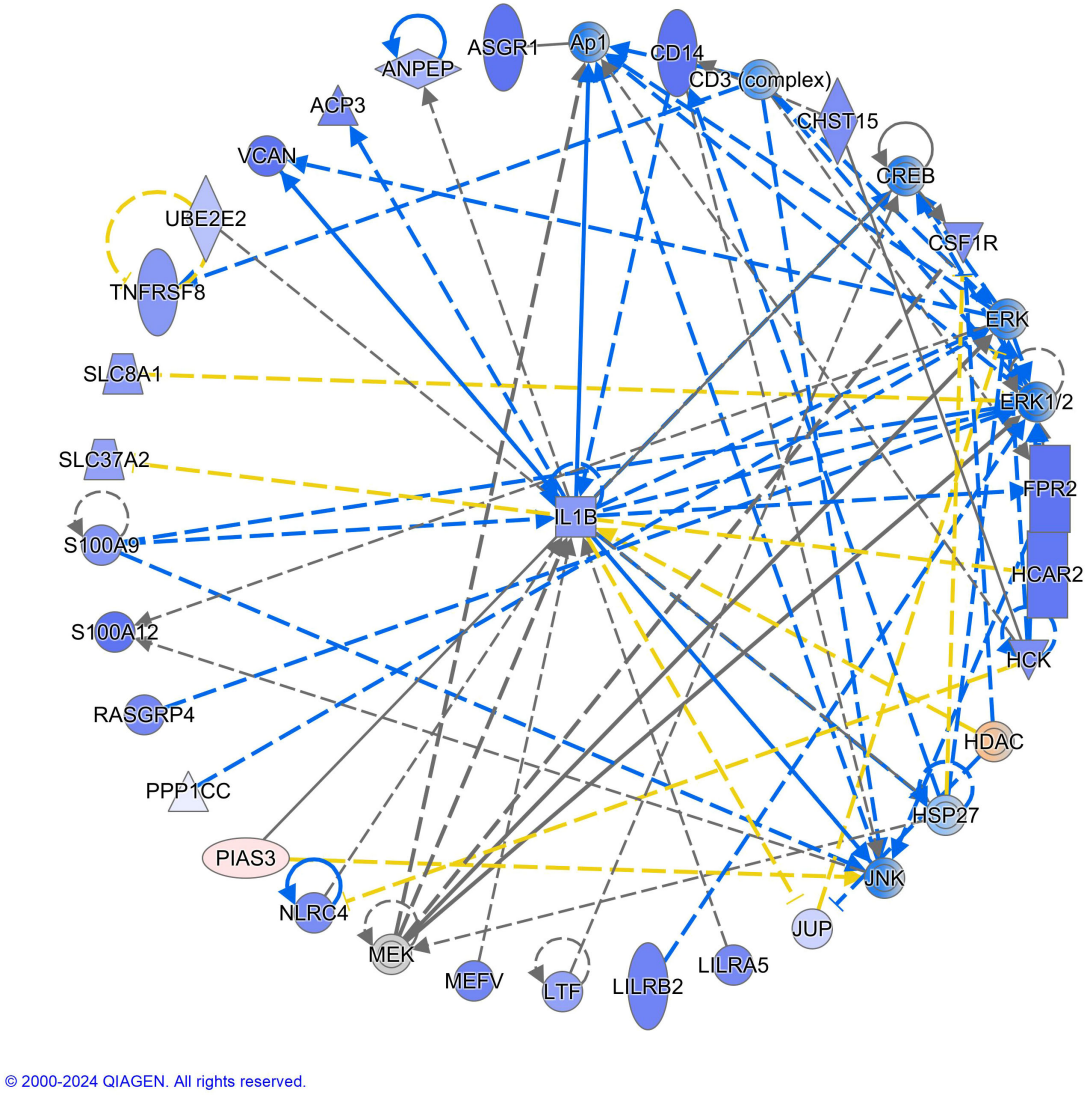
*IL1B* (Interleukin-1beta) was identified as the top-hub gene in the interactive network of the differential NK transcriptome response to acute exercise in children with acute lymphoblastic leukemia in remission compared to controls. The purple color of the nodes indicates negative log_2_FC values in the interaction analyses and the red color positive values. Blue and orange color for molecules indicates predicted inhibition and activation respectively. Molecule shapes and relationship lines are available in IPA®, QIAGEN Redwood City, www.qiagen.com/ingenuity. Solid and dashed lines reflect direct and indirect relationships between molecules. Circles lines show that the molecule interacts with itself (e.g., dimerization). The blue color of the arrows indicates “leads to inhibition,” orange “leads to activation,” yellow “findings inconsistent with the state downstream molecule,” and grey “effect not predicted.” Please see: https://qiagen.secure.force.com/KnowledgeBase/articles/Basic_Technical_Q_A/Legend for more details about molecule shapes and relationship.

Genes differentially affected by exercise in children with ALL in remission compared to controls [179 genes, log_2_FC values from interaction analyses ranged from -3.62 to 2.35 (163 genes showed negative values and 16 positive values); p < 0.01, (**Supplementary Table S3**)] were enriched in 28 gene pathways involved in inflammatory response and cancer disease (e.g., S100 Family Signaling Pathway, TREM1 Signaling, FAK Signaling, Molecular Mechanisms of Cancer, Breast Cancer Regulation by Stathmin1, Pathogen Induced Cytokine Storm Signaling Pathway, Toll-like Receptor Cascades) [FDR < 0.05, (**Figure 4**, **Table 3**)]. These pathways were inhibited by exercise in NK cells from cancer in remission compared to controls (Z-score values of activation ranged from -1.63 to -5.00, except for one pathway, “Cellular Effects of Sildenafil,” which was activated, Z-score = 3.36) (**Figure 4**, **Supplementary Table S4**). We did not observe a significant gene overlap between baseline differences and exercise effects between groups. The top-IPA gene network (IPA score 46) highlighted molecular functions, such as cellular function and maintenance, hematological system development and function, and inflammatory response differently altered by exercise in children with ALL in remission compared to controls (**Figure 5**), where *IL1B* gene presented the highest number of connections in this gene network (**Supplementary Table S5**). In addition to the results of interaction analyses (i.e., between-group comparisons; **Supplementary Table 3**), we reported within-group gene expression changes in children with ALL in remission and controls (**Supplementary Tables S6**, **S7**, **Supplementary Figure S1**).

**Table 3:** Selected gene pathways differently altered by exercise in natural killer (NK) cells from children with B-cell acute lymphoblastic leukemia (ALL) in remission compared to controls (FDR<0.05).

Gene pathways (number of genes in the pathway)	Link to inflammation, NK cell function or cancer	Genes identified in the pathway
Immunoregulatory interactions between a Lymphoid and a non-Lymphoid cell (12 genes)	This pathway was considered a therapeutic target for immune-based therapy (38).	*CD300E, CD300LF, CD34, JAML, LILRA1, LILRA4, LILRA5, LILRA6, LILRB2, LILRB3, LILRB4, TREM1*
S100 Family Signaling Pathway (20 genes)	S100 genes/proteins are involved in tumors’ development and drug resistance (40), as well as inflammatory responses and NK cell activation (43, 45).	*ADGRE1, ADGRE2, ADGRE3, C5AR1, CYBB, FPR1, FPR2, FZD1, GPR162, HCAR2, HCAR3, HTR7, IL1B, MMP17, NCF2, P2RY13, P2RY2, S100A12, S100A9, VIPR1*
TREM1 Signaling (5 genes)	It is involved in acute inflammatory responses against pathogens (51, 52) and represents an attractive therapeutic target for cancer (53).	*IL1B, NLRC4, TLR2, TLR8, TREM1*
FAK Signaling (16 genes)	FAK protein promotes tumor growth and metastasis (66). FAK expression in myeloid cells modulates the recruitment and survival of NK cells within the tumor microenvironment (66).	*ADGRE1, ADGRE2, ADGRE3, C5AR1, FPR1, FPR2, FZD1, GPR162, HCAR2, HCAR3, HTR7, P2RY13, P2RY2, TRAV8-3, TRGV5, VIPR1*
Toll-like Receptor Cascades (3 genes)	These receptors induce innate immune responses against pathogens, increasing NKCA and cytokine production (67).	*CD14, TLR2, TLR8*
Pathogen Induced Cytokine Storm Signaling Pathway (10 genes)	The overproduction of cytokines (i.e., cytokine storm) is observed in cancer disease and COVID-19 (68).	*CLEC7A, CSF2RA, CXCL16, CXCR3, IFNGR2, IL1B, NLRC4, SPI1, TLR2, TLR8*
Immunogenic Cell Death Signaling Pathway (5 genes)	Immunogenic Cell Death can trigger or boost immune responses against cancer (69).	*FPR1, IFNGR2, IL1B, LRP1, P2RY2*
G-Protein Coupled Receptor Signaling (14 genes)	G protein-coupled receptors might regulate NKCA and cytokine production, while the up-regulation of these proteins was associated with shorter overall survival in patients with acute myeloid leukemia (70).	*ADGRE1, ADGRE2, ADGRE3, C5AR1, FPR1, FPR2, FZD1, GPR162, HCAR2, HCAR3, HTR7, P2RY13, P2RY2, VIPR1*

FAK, focal adhesion kinase; NK, Natural killer; NKCA, NK-cell cytotoxic activity; TREM1, Triggering Receptor Expressed on Myeloid Cells 1.

## 4 Discussion

The main findings of this study were: i) at baseline and post-exercise, NKCA (*in vitro*) was lower following IL-2 activation in children with ALL in remission compared to controls, and several genes and pathways involved in NK cell function were differently expressed in circulating NK cells between the two groups; ii) an acute exercise session induced a comparable, robust (~two- to threefold) mobilization of NK cells into the peripheral circulation in the two groups; iii) NKCA (without IL-2 activation) following the acute exercise session significantly increased in both groups with no between-group differences; and iv) the immune gene expression pathway response to acute exercise differed between the two groups, as well as the expression of genes involved in inflammation and leukemia.

At baseline, children with ALL in remission exhibited lower NK cell function after IL-2 activation compared to controls. Additionally, we detected several genes that were differently expressed in circulating NK cells between groups (e.g., *HAVCR2*, *IL3RA*, *INPP5J, HLA-DQA1*, *IFNLR1, IL4, IFNLR1, ITGAD*, *ITGB8*), which are involved in key signaling pathways (IL-2 family signaling, glucocorticoid receptor signaling, and PI3K/AKT signaling). These differences may contribute to the decreased NK cell function after IL-2 activation (31–33). The IL-2 family signaling pathway is crucial for NK cell activation and proliferation. Altered gene expression in this pathway, such as *IL3RA* and *IL4*, may reduce NK cell response to IL-2 in children with ALL in remission (34). IL4 is a cytokine that can also interact with the glucocorticoid receptor signaling pathway, which exerts immunosuppressive effects that can diminish NK cell function. Additionally, the PI3K/AKT pathway, which is essential for cell survival and growth, can affect NK cell function through gene expression changes, such as *INPP5J*, impacting their survival and activation in response to IL-2. The gene pathway “Transcriptional Regulation by RUNX2” was activated at baseline in NK cells of ALL in remission compared to controls. It was observed that RUNX2 drives the generation of mature human NK cells (35) and regulates leukemic cell metabolism and chemotaxis in patients with T-cell ALL (36). These gene expression differences observed between groups could partially underlie the lower NKCA of ALL in remission compared to controls.

Previous studies in healthy adults have shown that a single bout of acute exercise increases NK cell numbers and NKCA (17, 22), as well as the expression of genes involved in the NK cell signaling pathway (24, 25). In our study, NK cell proportions, NKCA, and the expression of NK cell signaling pathways after acute exercise increased in both ALL and control groups in a similar magnitude (**Supplementary Figure S1**). Our results could overall suggest that acute exercise transiently improves immunosurveillance in ALL children in remission. Due to the capacity of an acute exercise stimulus to enhance the release of immune cell effectors, it has been suggested that immune cells from blood collected after an acute exercise bout might be used in the future as an adjunctive cell therapy for blood malignancies (17). Previous targeted-proteomics analyses have shown that regular exercise (16-week training) decreases systemic inflammation in survivors of pediatric B-cell ALL (19). In our study, an acute exercise bout induced a down-regulation of hundreds of genes and inhibited several gene pathways involved in inflammatory responses (**Figure 4**) in children with ALL in remission compared to controls (**Table 3**, **Figure 4**). The control group showed a higher expression of genes involved in the antigen processing and presentation pathway in NK cells after exercise (24, 25), suggesting an improved immunosurveillance (37). We observed that acute exercise reduced the expression of genes involved in the MHC I-mediated antigen processing and presentation pathway (*ASB13*, *CD14*, *CTSL*, *CYBB*, *NCF2*, *RNF144B*, *S100A9*, *TLR2*, *UBE2E2*) in the NK cells from children with ALL in remission, but not in the controls (**Supplementary Tables S6**, **S7**). The pathway “immunoregulatory interactions between a lymphoid and non-lymphoid cell” involves a number of cell surface receptors (e.g., NK cell inhibitory receptors [KIRs] and leukocyte immunoglobulin-like receptors [LILRs]) and cell adhesion molecules that are involved in the regulation of anti-tumor immunity (https://reactome.org/content/detail/R-HSA-198933). Shen et al. reported that several genes in the aforementioned pathway (highly enriched in all colorectal tumor locations and stages) were associated with survival and cytotoxic lymphocyte infiltration, suggesting this pathway might serve as a therapeutic target for immune-based therapy (38). In our study, we found that acute bouts of exercise inhibited this pathway in NK cells from children with ALL in remission compared to controls.

The ‘S100 family signaling pathway’ was inhibited by exercise in the children with ALL in remission compared to controls. S100 genes/proteins (expressed in immune effectors such as CD8^+^ T and NK cells) (39) belong to a family of calcium-binding proteins with important roles in tumors’ development and drug resistance (40). Some pro-inflammatory molecules in the S100 family signaling pathway, such as S100A8, S100A9, and S100A12, have been considered targets to improve treatment efficiency in ALL (41, 42). In our study, the expression of *S100A8*, *S100A9, and S100A12* genes were reduced by acute exercise in the NK cells from the patients in remission, with no change found in their controls (**Supplementary Tables S6**, **S7**). S100A8, S100A9, and S100A12 interact with toll-like receptors and G protein-coupled receptors [these pathways were differently altered by exercise in ALL in remission compared to controls, (**Figure 4**, **Table 3**)] to regulate inflammatory responses in innate immune cells (43, 44), while a higher S100A8 and S100A9 protein expression in NK cells induces their activation (45). Furthermore, S100A8, S100A9, and S100A12 proteins act as sensors of intracellular Ca^2+^ levels (44), which is important for the mobilization of NK cells as well as for NKCA (46, 47). The exercise-induced downregulation of the S100 family signaling pathway may reflect a reduction in inflammatory priming of NK cells post-exercise in children with ALL in remission. This may represent a protective mechanism against chronic low-grade inflammation often seen in cancer survivors. Given the involvement of S100A8 and S100A9 in leukemic progression and treatment resistance (48–50), these molecular changes highlight the possible therapeutic potential of acute exercise in modulating pro-inflammatory mediators in children with ALL in remission.

TREM1 signaling pathway was inhibited by exercise in NK cells of children with ALL in remission compared to controls, and the *TREM1* gene showed one of the highest down-regulation patterns induced by exercise (top-10 log_2_FC values) in the former while it was not altered by exercise in controls (**Supplementary Tables S6**, **S7**). *TREM1* encodes a transmembrane glycoprotein receptor that is mainly expressed in neutrophils, monocytes, tissue macrophages, and NK cells (51, 52), which is important for the triggering and amplifying acute inflammatory responses against pathogens (51, 52). Additionally, a recent study showed that TREM1 plays a role in the inflamed tumor microenvironment, and its inhibition enhances the antitumorigenic effect of anti–PD-1 treatment in murine melanoma, representing an attractive therapeutic target for cancer (53). Interestingly, one study showed that higher physical activity levels were associated with the inhibition of the TREM1 signaling pathway in whole blood from adults with rheumatoid arthritis (54). Similarly, a 12-month home-based exercise training intervention reduced *TREM1* expression in monocytes, as well as the soluble TREM1 concentration in plasma from adults with intermittent claudication (55). The reduced TREM1 expression level in NK cells from children with ALL in remissions immediately following an acute bout of exercise may indicate broader anti-inflammatory reprogramming of the immune response, and/or reflect a transient shift in NK cell subpopulations. This overall decrease in circulating TREM1 expression could potentially contribute to reduced systemic inflammation and improved immune regulation. Given TREM1’s emerging role in tumor progression and immune escape mechanisms, its modulation by exercise could support improved immune surveillance and enhance responsiveness to immunotherapeutic strategies. These findings offer new insights into how exercise may serve as a non-pharmacological adjunct to restore immune competence in children with ALL in remission.

*IL1B* could represent an important molecular driver of the differential transcriptome response to exercise observed in the group of children with ALL in remission compared to their controls; however, these gene expression changes may also result from exercise-induced shifts in NK cell subpopulation composition. *IL1B* was reduced after acute exercise in NK cells of children with ALL in remission but not in controls (log2FC -1.68 *vs*. -0.18; p<0.001 and p=0.59 respectively, [**Supplementary Tables S6**, **S7**) and it was the gene with the higher number of connections within the interactive gene network enriched in inflammatory response (**Figure 5**). IL-1β is a well-known pro-inflammatory cytokine (56), and *IL1B* gene expression levels at diagnosis predict the relapse in children with pre-B-cell ALL (57). It could be expected a higher expression of *IL1B* gene in NK cells after acute exercise (i.e., inflammatory response), but findings on this topic are inconsistent in PBMCs (58). For example, circulating IL-1β increased after acute exercise but the latter did not change *IL1B* gene expression in PBMCs from the same healthy young men (59). It has been reported that IL-1β limited the level of NK cell activation by causing these cells to undergo apoptosis (60), while we observed that acute exercise reduced the expression of the gene encoding this cytokine in NK cells from children with ALL in remission but not in controls. Future studies should compare the impact of acute exercise on *IL1B* gene and protein expression in different leukocyte populations and types of cancers, as well as long-term exercise interventions, to reveal the clinical implications of these findings. In summary, the NK cell transcriptome profile showed a differential inflammatory response to acute exercise in children with ALL in remission compared to their controls, which could be related to anti-tumor immunity.

The changes in NK cell gene expression following exercise are driven by a complex interplay of hormonal, neural, and cytokine-mediated signals, as well as direct cellular signaling mechanisms. In our study, most of the children with ALL in remission and healthy controls were male. In this regard, it is known that hormonal differences in males and females during growth can affect NK responses to acute exercise with greater responses in the latter (21). Future studies should expand our findings by considering sex-specific responses. There is also evidence that anti-tumor activity of NK cells may also depend on specific pattern of marginating pools (61). These changes help to enhance the functional capacity of NK cells and contribute to the overall benefits of exercise on immune health (17, 18). The current study design cannot elucidate whether the changes induced in the gene expression of circulating NK cells would also apply to those located in the different bodily reservoirs outside the bloodstream (e.g., NK cells attached to the vascular endothelium, or resident in bone marrow or liver) (24). Additionally, a fixed amount of PBMCs (including NK cells, monocytes, and cytotoxic CD8+ T cells) was used for co-culturing with the target cells to assess specific NKCA. Since acute exercise increases the relative abundance of NK cells, monocytes and cytotoxic CD8+ T cells in peripheral blood and the PBMCs fraction (17), we cannot determine whether the increased NKCA after exercise is due solely to enhanced NK cell activity, a higher proportion of NK cells, or potential contributions from other cytotoxic subsets such as monocytes and cytotoxic CD8+ T cells. Future studies may employ spectral flow cytometry to characterize NK cell subtypes, monocytes and CD8+ T cells more precisely, plasma proteomics to assess a broad panel of cytokines, single-cell omics approaches (e.g., scRNA-seq, ATAC-seq) to uncover regulatory mechanisms and cellular heterogeneity not captured by bulk RNA-seq. Together, these complementary methods would deepen mechanistic insight into immune cell dynamics in response to acute exercise in children with ALL in remission.

The exercise protocol was identical in both groups in terms of duration and intensity, inducing a similar increase in blood lactate, NK cell proportions, and relative increase in NKCA *in vitro* after exercise. Nevertheless, *in vitro* NKCA may not directly translate to *in vivo* anti-tumor efficacy, particularly in immunocompromised hosts. The distinct immune gene pathway activation pattern in response to exercise found in ALL children in remission suggests that their NK cells might adopt a different molecular strategy to fight against infections or tumors. Our results identify candidate genes and molecular pathways through which exercise may benefit the immune health status. These findings align with NK cell functional data from this study and previous research on inflammatory proteins (19), supporting exercise as a potential non-pharmacological intervention for children with ALL in remission. We applied a stringent p-value cutoff of 0.01 for RNA-seq differential expression analysis and used false discovery rate (FDR) correction to account for multiple comparisons in canonical pathway analysis, enhancing result reliability. However, the small sample size, determined by feasibility reasons, limits the generalization of our transcriptome findings, which are hypothesis-generating and lay the groundwork for future larger-scale investigations. Additionally, we did not have access to participants’ electronic medical records and therefore lacked information on B-cell ALL subtypes and treatment regimens. Future exercise studies using larger sample sizes, randomized controlled designs, and multi-omics (including single-cell) analyses (62–65) are warranted in this and other pediatric cancer-related populations. Understanding the molecular changes induced by exercise can help in designing exercise programs that optimize immune function, particularly for populations with compromised immunity.

## 5 Conclusions

Distinct immune gene pathway responses to acute exercise were observed in circulating NK cells from B-cell ALL children in remission compared to controls with no history of cancer. Studying the NK-cell genomic response to exercise could thus be helpful in identifying molecular pathways and candidate genes related to impaired NK cell function or differential inflammatory response to physical stress after surviving cancer, which might remain unnoticed if studying solely baseline (i.e., non-exercise) conditions. Although NKCA following IL-2 stimulation was lower at baseline and post-exercise in the children with ALL in remission compared to controls. In both groups, acute exercise induced a similar relative increase in unstimulated NKCA. Therefore, while further research is needed, exercise might be considered to improve NK cell function in children with ALL in remission.

## Data Availability

The gene data analyzed in this article have been deposited in the Gene Expression Omnibus. Gene expression GEO record: GSE272928 (https://www.ncbi.nlm.nih.gov/geo/query/acc.cgi?acc=GSE272928).

## Supplementary material

Supplementary Figure 1 Within-group comparison of gene expression analysis (i.e., post- vs. preexercise, separately in each group) showed gene pathways altered by exercise in children with acute lymphoblastic leukemia (ALL) in remission and controls (CONT). The blue color represents inhibition and orange activation, and the gray effect is not predicted.
