# Supplementary figures and images for: Distinct NK cell function and gene expression in children with acute lymphoblastic leukemia in remission before and after acute exercise: an exploratory study
Source: Front Immunol. 2025 Aug 13;16:1625437. doi: 10.3389/fimmu.2025.1625437 (PMC12380555; doi:10.3389/fimmu.2025.1625437)

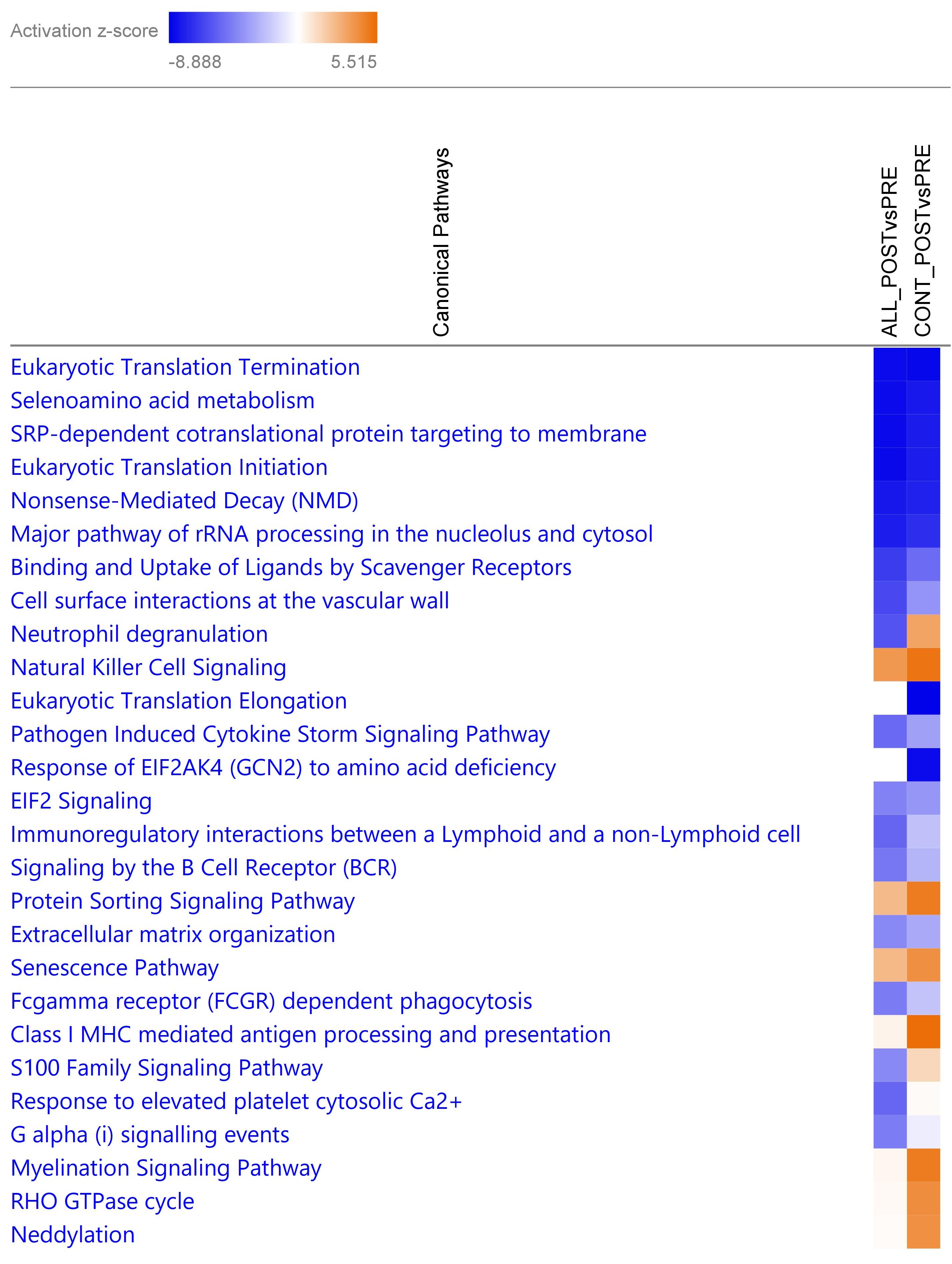

Supplement: Supplementary Figure 1 — Within-group comparison of gene expression analysis (i.e., post- vs. preexercise, separately in each group) showed gene pathways altered by exercise in children with acute lymphoblastic leukemia (ALL) in remission and controls (CONT). The blue color represents inhibition and orange activation, and the gray effect is not predicted. [file Image1.jpeg]
